# Functional Characterization of Plant Peptide-Containing Sulfated Tyrosine (PSY) Family in Wheat (*Triticum aestivum* L.)

**DOI:** 10.3390/ijms252312663

**Published:** 2024-11-25

**Authors:** Peipei Zhang, Weidong Gao, Lijian Guo, Ming Chen, Jingfu Ma, Tian Tian, Yanjie Wang, Xiwei Zhang, Yongtong Wei, Tao Chen, Delong Yang

**Affiliations:** 1 State Key Laboratory of Aridland Crop Science, Gansu Agricultural University, Lanzhou 730070, China; freedom313514@163.com (P.Z.); 15294208264@163.com (W.G.); guolj@gsau.edu.cn (L.G.); 2 College of Life Science and Technology, Gansu Agricultural University, Lanzhou 730070, China; cm22882122@163.com (M.C.); 18294180511@163.com (Y.W.); zhangxw20002024@163.com (X.Z.); ct2545@126.com (T.C.); 3 College of Agronomy, Gansu Agricultural University, Lanzhou 730070, China; majf@st.gsau.edu.cn (J.M.); t18893911748@163.com (T.T.)

**Keywords:** wheat gene family, PSY peptide, expression pattern, root growth

## Abstract

The plant peptide-containing sulfated tyrosine (PSY) family plays critical roles in plant cell proliferation and stress responses. However, the functional characterization of the PSY peptide family in wheat remains unclear. This study systematically identified a total of 29 *TaPSY* genes at the genome-wide level, classifying them into six subgroups based on PSY-like motifs. These peptides contain a highly conserved active peptide domain, closely resembling the *Arabidopsis* AtPSY1 motif. All TaPSY homologs are predicted to have a sulfated tyrosine catalyzed by plant tyrosylprotein sulfotransferase (TPST). The *TaPSY* genes displayed distinct expression patterns across various tissues, with most genes showing higher expression levels in roots and stems. Synthetic sulfated TaPSY peptides enhanced root growth in both wild-type *Arabidopsis* and the *tpst-1* mutant plants. In wheat, exogenous application of TaPSY peptides also promoted root growth, with the synthetic TaPSY5 peptide affecting reactive oxygen species levels in wheat taproots to stimulate primary root growth. Furthermore, transgenic *Arabidopsis* plants overexpressing *TaPSY10* exhibited longer primary roots and increased lateral root numbers. These findings provide insights into the physiological roles of TaPSY peptides in regulating wheat root growth.

## 1. Introduction

Small peptides are vital regulators of cell-to-cell signaling, influencing plant growth, development, and stress responses [1]. Over the last two decades, more than 30 classes of plant peptide hormones have been identified, such as phytosulfokine (PSK), Casparian strip integrity factors (CIFs), rapid alkalinization factor (RALF), and root growth meristem factors (RGF1) [2,3,4,5,6,7]. These peptides, ranging from 2 to 100 amino acids, are derived from non-functional prepropeptide precursors that undergo proteolytic cleavage and post-translational modifications (PTMs) to become biologically active [8]. Among these peptides, the Tyr-disulfated pentapeptide (PSK) and plant peptide-containing sulfated tyrosine (PSY) stimulate cell expansion and division at nanomolar concentrations [9]. PSK, a disulfated YIYTQ pentapeptide (Tyr(SO_3_H)-Ile-Tyr(SO_3_H)-Thr-Gln), is involved in root and hypocotyl elongation, somatic embryogenesis, and immunity response regulation [10,11,12]. While the roles of PSK are well studied across various species, research on PSY peptides remains limited.

Nine members of the PSY family have been identified in *Arabidopsis thaliana*, with AtPSY1 being the most extensively characterized. The peptide PSY1 is an 18-amino acid tyrosine-sulfated glycopeptide that promotes cell proliferation at low concentrations [13]. The mature PSY1 peptide contains three PTMs: a sulfated tyrosine, two hydroxyproline residues, and a tri-L-arabinose side chain [13]. It has been previously demonstrated that the sulfation of the tyrosine residue in PSY1 is necessary for its full biological activity, and this modification is mediated by tyrosylprotein sulfotransferase (TPST) in *Arabidopsis*, localized in the trans-Golgi apparatus. TPST loss-of-function mutants exhibit shorter roots and lower biomass [14].

The PSY polypeptide precursors are encoded by a small group of nuclear genes in plants. The *PSY* gene family has been identified in some plant species, such as *Arabidopsis* with nine members and rice (*Orysa sativa* L.) with seven members [15]. *AtPSY1* plays a crucial role in root and hypocotyl development, with its overexpression leading to longer roots and hypocotyls [13]. Further research has revealed that PSY1 is recognized by the leucine-rich receptor kinase PSY1R (LRR-RLK PSY1 RECEPTOR), which subsequently interacts with and phosphorylates the plasma membrane-localized H^+^-ATPases AHA1 and AHA2. This interaction leads to cell wall acidification, thereby promoting cell elongation and tissue growth [16]. Pruitt et al. (2017) reported that a bacterial pathogen peptide, RaxX, derived from *Xanthomonas oryzae* pv. *oryzae* (*Xoo*), shares high sequence similarity with the AtPSY1 peptide [17]. RaxX acts as a virulence factor that facilitates *Xoo* infection by inducing the expression of defense genes, which are essential for the activation of the XA21-mediated plant immune response [18]. A synthetic sulfated RaxX peptide (RaxX13-sY) has been shown to promote root growth in both rice and *Arabidopsis* [17]. Furthermore, a previous report showed that two sulfated peptides, PSY1 and CIF2, secreted by the endosperm, promote cuticle formation in *Arabidopsis* during the transition from embryo to seedling. Mutations in *psy1* or *psy1r* impair cuticle formation, underscoring their crucial role in this process [19]. Recent research by Ogawa-Ohnishi et al. (2022) has shown that PSY family peptides bind to three leucine-rich repeat receptor kinases (PSYR1, PSYR2, and PSYR3) to regulate plant growth and stress responses [20,21]. PSY peptides maintain normal plant growth by inhibiting the expression of stress response genes, while *PSYRs* activate stress tolerance-related genes in the absence of ligands [20]. Although the *PSY* family genes have been well characterized in *Arabidopsis*, their roles and potential functions in wheat remain unknown.

This study systematically investigated the wheat PSY peptide family using bioinformatics tools. We examined evolutionary relationships, gene structures, conservative motifs, *cis*-acting elements, chromosomal locations, gene duplication events, tissue expression patterns, and responses to environmental signals. A total of 29 *TaPSY* genes were identified, all of which share a conserved PSY-like motif in their C-terminal region. The exogenous application of synthetic TaPSY peptides enhanced primary root growth in both wild-type *Arabidopsis* and wheat seedlings. Moreover, the *TaPSY10* gene exhibited the highest expression levels in the root, and overexpression of *TaPSY10* in *Arabidopsis* led to increased primary root growth and lateral root number. These findings provide valuable insights into the role of small regulatory peptides during plant root development.

## 2. Results

### 2.1. Genome-Wide Identification and Characterization of the TaPSY Gene Family

**Figure 1:**
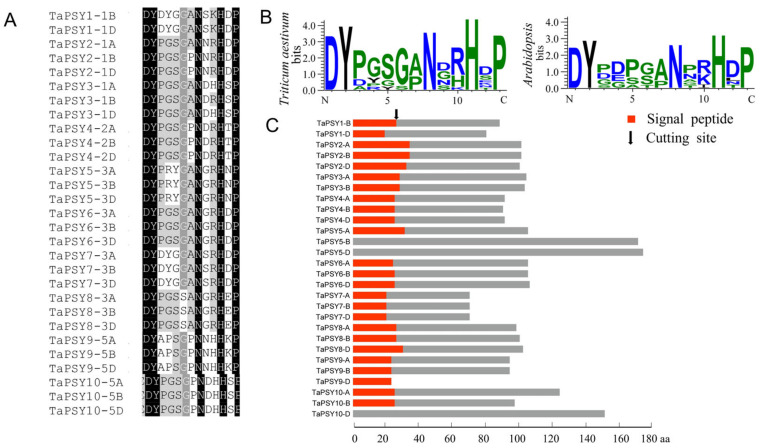
Identification of the *TaPSY* gene family. (**A**) Alignment of the 29 TaPSY motifs. (**B**) WebLogo represents the conserved TaPSY and AtPSY motifs. (**C**) Signal peptides of TaPSY proteins. The red boxes represent signal peptides, and the black arrows represent the predicted signal peptide cleavage sites.

PSY precursor protein sequences from *Arabidopsis* and rice were used to perform a BLASTP search against the *Triticum aestivum* Chinese Spring genome database. Query sequences containing the conserved PSY-like motif (DYXXXX[AP]NXXHXP), similar to the AtPSY domain sequence from *A. thaliana*, were selected. As a result, 29 *TaPSY* genes were identified in the latest released wheat genome (Figure 1A,B). The detailed characteristics of the identified TaPSY proteins are listed in Table S2. The TaPSY precursors have varying physicochemical properties and amino acid sequences, with lengths ranging from 71 amino acids (TaPSY7-3A/3B/3D) to 175 amino acids (TaPSK5-3D). The molecular weights of the TaPSY proteins vary from 7873.08 Da (TaPSY7-3B) to 19,355.04 Da (TaPSK5-3D), and the isoelectric points (pI) range from 4.98 (TaPSY6-3A) to 11.8 (TaPSK2-1A). Subsequently, the analysis of N-terminal signal peptides in each TaPSY protein using TargetP 2.0 and iPSORT revealed that, with the exception of TaPSY5-3B, TaPSY5-3D, and TaPSY10-5D, all other TaPSY family members contain signal peptide sequences ranging from 20 to 35 amino acids (Figure 1C).

**Figure 2:**
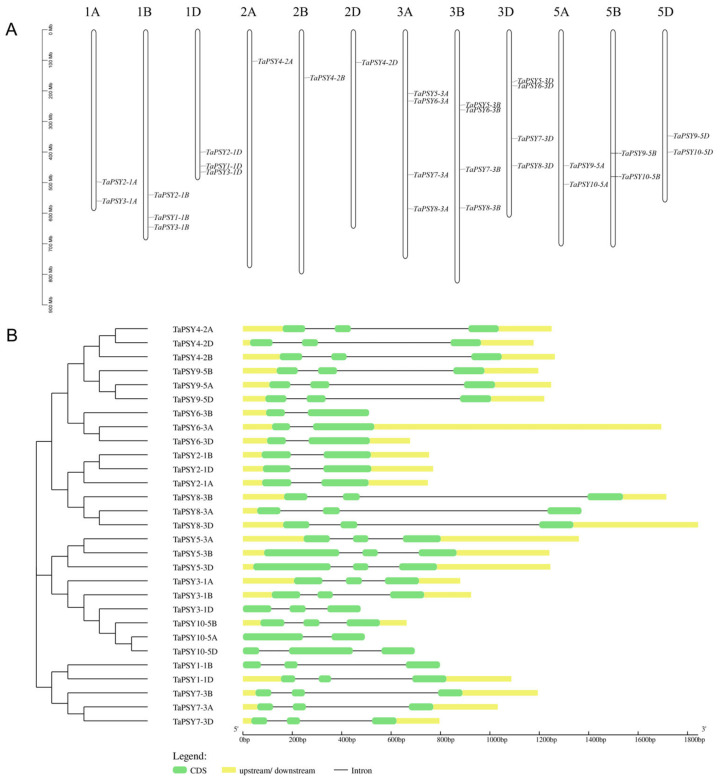
Chromosome localization and gene structures of the *TaPSY* genes. (**A**) Distribution of *TaPSY* genes on wheat chromosomes. (**B**) Gene structure of *TaPSY* genes. The green boxes and black lines represent exons and introns, respectively.

Chromosome localization analysis revealed that the putative *TaPSY* genes are unevenly distributed across 12 of the 21 chromosomes of the wheat genome, specifically mapped to homologous groups 1, 2, 3, and 5 (Figure 2A). Among these, chromosomes 3A, 3B, and 3D each contain four *TaPSY* genes, followed by chromosomes 1B and 1D, which harbor three *TaPSY* genes. Additionally, one *TaPSY* gene is located on chromosomes 2A, 2B, and 2D. As shown in Figure 2, three homologous copies of *TaPSY* clustered in the same branch have similar gene structures. However, structural differences exist in the number of exons and their positions among these *TaPSY* genes. Notably, *TaPSY2-1A/1B/1D*, *TaPSY6-3A/3B/3D*, and *TaPSY10-5A* genes are found to have two exons, while the remaining *TaPSYs* possess three introns (Figure 2B).

### 2.2. Phylogenetic Analysis and Duplication of TaPSYs

**Figure 3:**
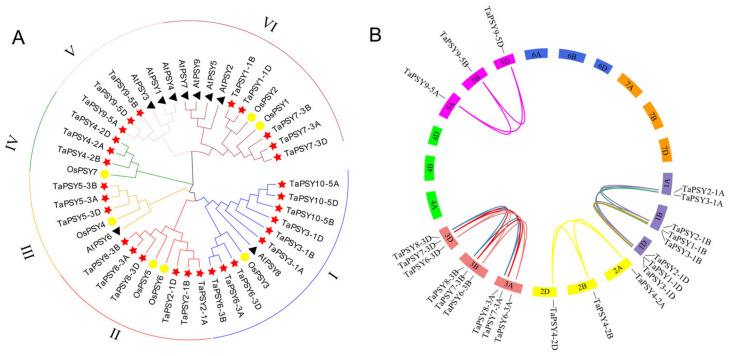
The phylogenetic tree of PSYs and segmentally duplicated gene pairs in the wheat genome. (**A**) The phylogenetic tree of TaPSYs from *Triticum aestivum*, *Arabidopsis thaliana*, and *Oryza sativa*. Clades with colored branches refer to different subfamilies. Amino acid sequences of all 45 PSY proteins were aligned using ClustalX 2.01 program. *Ta*, *Triticum aestivum*; *Os*, *Oryza sativa*; and *At*, *Arabidopsis thaliana*. The phylogenetic tree was constructed using MEGA7.0 software with 1000 bootstrap replicates. AtPSYs, OsPSYs, and TaPSYs are indicated by black triangles, yellow circles, and red stars, respectively. (**B**) The segmentally duplicated *TaPSY* gene pairs in the wheat genome. The chromosome columns of the same chromosome group are indicated by the same color, and different chromosome groups are distinguished by different colors. The duplicated gene pairs are linked using different colored lines.

To understand the evolutionary relationships of PSY proteins, we constructed a phylogenetic tree using a total of 45 PSY protein sequences, which included 29 TaPSYs, 7 OsPSYs, and 9 AtPSY proteins. Based on the alignment of the full-length protein sequences, the *PSY* genes were categorized into six subgroups (Groups I, II, III, IV, V, and VI), with each subgroup containing 11, 8, 5, 4, 6, and 11 PSY proteins, respectively. Among these groups, Groups I and VI harbor the highest number of genes, accounting for up to 60% of the identified genes. Notably, *AtPSY* genes in *A. thaliana* are mainly found in Groups V and VI, while Groups II and IV do not contain any *AtPSY* genes (Figure 3A).

In terms of segmental and tandem duplications, we identified 19 gene pairs involved in segmental duplication. However, no tandem-duplicated genes were detected in the wheat *TaPSY* gene family (Table S3). We observed that the majority of homologous gene groups (*TaPSY2*-*TaPSY10*) have one copy on each of the A, B, and D homologous chromosomes, while *TaPSY1* possesses two copies located on chromosomes 1B and 1D (Figure 3B). To further analyze the selective constraints on the *TaPSY* gene family, the Ka/Ks ratios of duplicated gene pairs were calculated. The results show that the Ka/Ks ratios for most paralogous genes are less than 1, ranging from 0.069 to 0.648, suggesting that they have been subjected to purifying selection during the evolutionary process. Notably, the Ka/Ks ratio of the duplicated gene pair *TaPSY2-1B/TaPSY2-1D* is > 2, indicating strong positive selection pressure on this gene pair. In contrast, the Ka/Ks ratio of the paralogous gene pair *TaPSY9-5A/TaPSY9-5B* is close to 1, suggesting that they may have undergone neutral selection during their evolution.

### 2.3. Multiple Alignment and Conserved Domain Analysis of TaPSYs

Multiple sequence alignment revealed that all TaPSY homologs share a conserved 13-amino acid PSY-like motif (DYXXXX[AP]NXXHXP) in the C-terminal region, corresponding to the active domain of AtPSY1, where ‘X’ represents any amino acid. Despite this conserved region, TaPSY precursor sequences exhibit significant divergence at the N-terminus. Except for TaPSY5 and TaPSY10, the other homologous copies of precursor peptides exhibit similar lengths, with over 80% identity in their amino acid sequences (Figure S1).

### 2.4. Cis-Acting Regulatory Elements in TaPSY Promoters

Analysis of cis-acting elements in the promoter region of *TaPSY* genes identified several *cis*-regulatory elements. Beyond the TATA box and light-responsive elements, we found 13 plant hormone-responsive elements, 15 elements related to biotic and abiotic stress responses, and 8 elements involved in plant growth and development (Figure S2). Notably, the ABA-responsive element (ABRE) was distributed in 86% (25 of 29) of *TaPSY* genes, while MeJA-responsive elements (CGTCA/TGACG motifs) appeared in several *TaPSY* genes. Additionally, auxin response elements (AuxRR core, TGA element) and ethylene response elements (ERE) were detected in some *TaPSY* gene promoters. Gibberellin-responsive elements (P-box) were found in the three homoeologs of *TaPSY7* genes. Stress-related cis-acting elements included drought-response elements (MBS, MYC, MYB recognition site, W-box, DREcore), low-temperature response elements (LTR), and defense and stress-responsive elements (TC-rich repeats). Notably, the MBS element (MYB binding site involved in drought inducibility) was widely distributed in 62% (18 of 29) of *TaPSY* genes. Furthermore, growth-related elements included the meristematic expression elements (CAT box and CCGTCC motif), gliadin metabolic element (GCN4_motif), seed-specific regulation element (Ry-element), and corn gliadin metabolic regulatory element (O2 site).

### 2.5. Expression Patterns of TaPSY Genes in Different Tissues

RNA-seq data analysis revealed tissue-specific expression of *TaPSY* genes. For instance, 13 *TaPSY* genes exhibited relatively high expression levels in roots, while 14 genes showed elevated expression levels in stems. *TaPSY3-1A/1B/1D*, *TaPSY4-2A/2B/2D*, and *TaPSY10-5A/5B/5D* displayed significantly higher expression in roots and stems, while *TaPSY9-5A/5B/5D* and *TaPSY6-3A/3B/3D* were predominantly expressed in stems, particularly during the jointing stage (Stem_Z32). Additionally, *TaPSY5-3B* was highly expressed in grain at the Z_71 and Z_85 stages (Figure S3).

**Figure 4:**
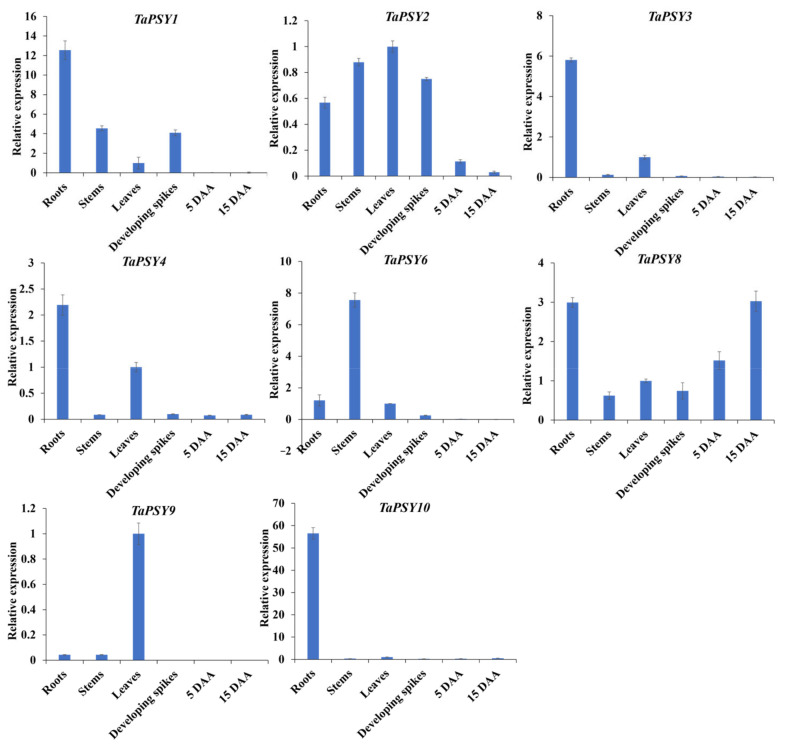
Expression profiling of *TaPSY* genes in various wheat tissues: roots, stems, leaves, developing spikes, and grains at 5 and 15 DAA. The gene expression in leaves was set as 1. Relative gene expressions were normalized to the reference gene *GAPDH* and calculated using the 2^-ΔΔCt^ method. Data represent the mean ± standard error of the mean (SEM) of three biological replicates.

To further explore expression in various tissues (seeds, leaves, roots, developing spikes, and grains at 5 DAA and 15 DAA), qRT-PCR was used to examine the spatial expression patterns of selected *TaPSY* genes. Given the short coding sequences, primers were designed to amplify three homologous genes at conserved regions. For example, the expression levels of *TaPSY3* represent the combined expression of all three homologous *TaPSY3* alleles (*TaPSY3-1A*, *TaPSY3-1B*, and *TaPSY3-1D*). The results revealed distinct expression patterns in the tested tissues (Figure 4). *TaPSY6* was predominantly expressed in stems, while *TaPSY9* showed the highest expression in leaves, and *TaPSY10* had the highest expression in roots. *TaPSY3* and *TaPSY4* were highly expressed in both roots and leaves. Except for *TaPSY8*, most *TaPSY* genes showed low or undetectable expression in grains. These findings suggest that *TaPSY* genes play specialized roles in tissue development.

### 2.6. TaPSYs Respond to Various Environmental and Hormonal Stimuli

The expression profiles of *TaPSY* genes were analyzed under abiotic stresses, such as drought, high temperature, their combination, and salt stress, using publicly available RNA-seq data. As shown in Figure S4A, the transcript levels of *TaPSY8-3A/3B/3D*, *TaPSY6-3B*, and *TaPSY1-1D* were significantly upregulated after 6 h of drought stress. The expression levels of *TaPSY2-1A/1B/1D* exhibited increases at both 1 and 6 h post-drought stress. In the case of temperature stress, approximately 50% of the affected genes, such as *TaPSY5-3A/3B/3D*, *TaPSY7-3A/3B/3D*, *TaPSY3-1D*, and *TaPSY9-5A/5B/5D*, were upregulated after 6 h of heat stress (HS). Under combined drought and heat stress (HD) conditions, the expressions of *TaPSY1-1B/1D*, *TaPSY3-1D*, *TaPSY7-3A/3B/3D*, *TaPSY8-3A*, and *TaPSY10-5A* were upregulated at 6 h following the stress treatment.

In response to salt stress, the transcriptional levels of seven genes, including *TaPSY6-3D*, *TaPSY8-3A/3B/3D*, and *TaPSY9-5A/5B/5D*, were significantly upregulated over time, while other genes such as *TaPSY2-1D*, *TaPSY3-1B*, *TaPSY6-3A,* and *TaPSY10-5A/5B/5D* were downregulated at various time points. *TaPSY4-2A/2B/2D* showed a consistent decrease in expression at 6 and 12 h (Figure S4B).

**Figure 5:**
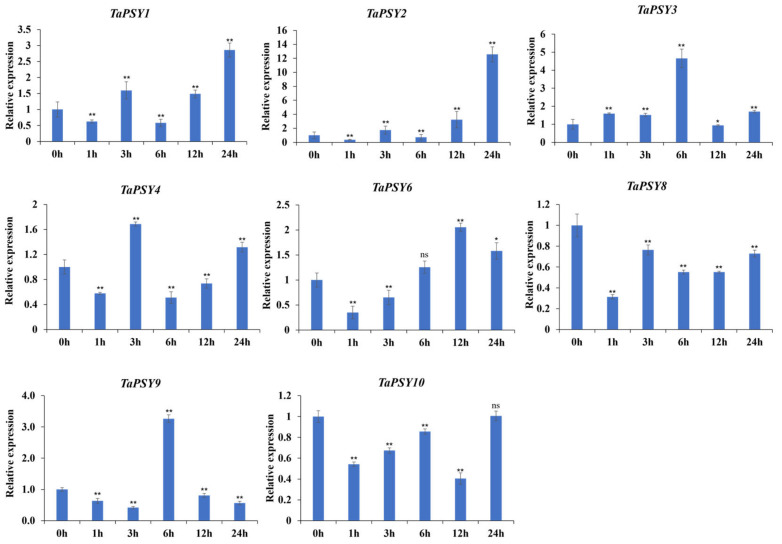
Expression levels of selected *TaPSY* genes in wheat roots under PEG-6000 treatment validated by qRT-PCR. Gene expression under control conditions was normalized to 1, with *TaActin* used as an internal control. Error bars represent the mean ± SEM of three biological replicates. Statistically significant differences between the control and treatment groups are indicated by asterisks. * *p* < 0.05 and ** *p* < 0.01; ns, non-significant difference.

Since most *TaPSY* genes are predominantly expressed in roots, we used qRT-PCR to assess transcription levels in 14-day-old Chinese Spring roots treated with 20% PEG6000, 100 µM ABA, and 100 µM IAA. The expressions of *TaPSY1* and *TaPSY2* were significantly upregulated after 24 h of drought stress, showing 2.8- and 12.5-fold changes relative to controls, respectively. In contrast, *TaPSY8* expression significantly decreased at all time points. *TaPSY3* showed increased transcription at 0–6 h after treatment, followed by a decline thereafter. *TaPSY4*, *TaPSY6*, and *TaPSY9* were upregulated at specific time points (Figure 5).

**Figure 6:**
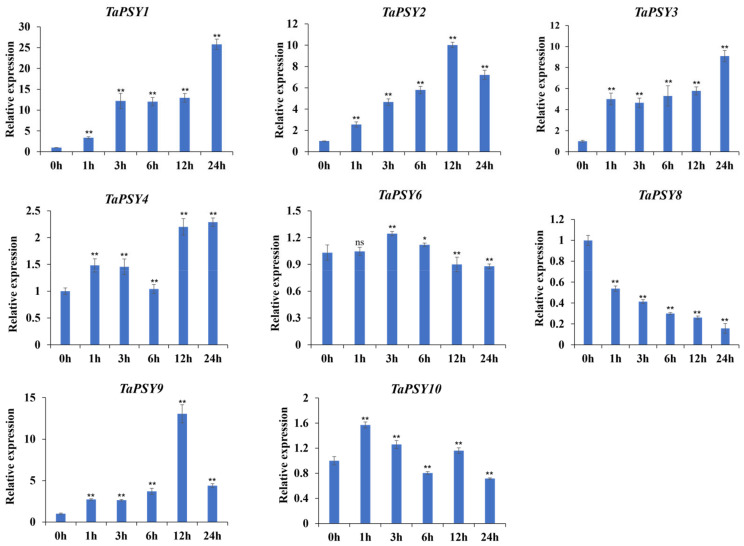
Expression levels of selected *TaPSY* genes in wheat roots under IAA treatment validated by qRT-PCR. Expression was normalized to 1 under control conditions, with *TaActin* used as an internal control. Error bars indicate the mean ± SE of three biological replicates. Statistically significant differences between the control group and treatment group are indicated by asterisks. * *p* < 0.05 and ** *p* < 0.01; ns, non-significant difference.

**Figure 7:**
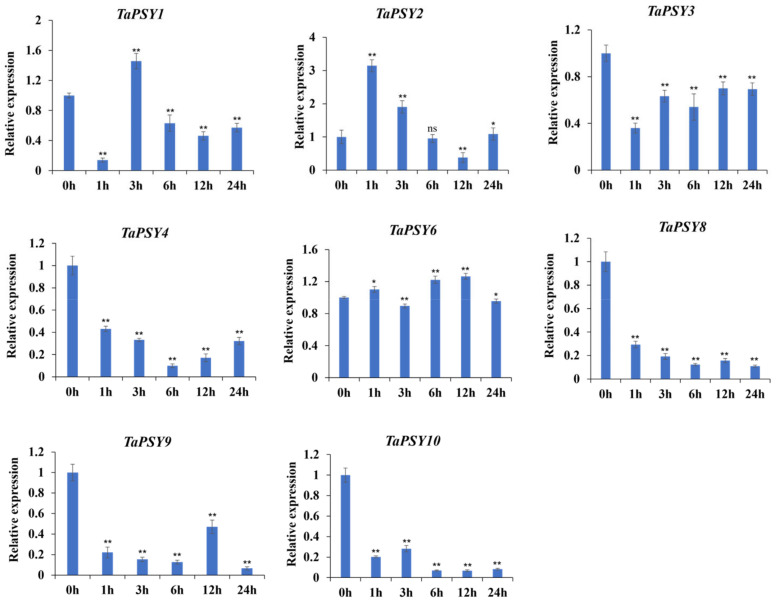
Expression levels of selected *TaPSY* genes in wheat roots under ABA treatment validated by qRT-PCR. The expression level of each gene under the control treatment was normalized to 1, with *TaActin* used as an internal control. Error bars indicate the mean ± SE of three biological replicates. Statistically significant differences between the control group and treatment group are indicated by asterisks. * *p* < 0.05 and ** *p* < 0.01; ns, non-significant difference.

Upon IAA treatment, the expression levels of *TaPSY1*, *TaPSY2*, *TaPSY3*, and *TaPSY9* were upregulated at all time points, while *TaPSY8* expression was significantly downregulated (from 0.14- to 0.54-fold) in roots exposed to IAA. Notably, the expression of *TaPSY4* showed a 2.2-fold increase after 12 h of IAA induction, whereas *TaPSY6* exhibited a more moderate response (Figure 6). Under ABA treatment, *TaPSY2* and *TaPSY1* reached peak expression at 1 h and 3 h, respectively, followed by a decrease in expression. In contrast, *TaPSY3*, *TaPSY8*, *TaPSY9*, and *TaPSY10* were significantly downregulated, with expression levels ranging from 0.11- to 0.69-fold compared to controls (Figure 7).

### 2.7. TaPSY Peptides Promote Primary Root Growth in Wheat and Arabidopsis

To evaluate the physiological effects of TaPSY peptides on root growth, we synthesized TaPSY4, TaPSY5, TaPSY7, and TaPSY8 peptides. Root growth assays were conducted using wild-type *Arabidopsis* and the *tpst-1* mutant, a T-DNA insertion line lacking the *AtTPST* gene. This gene encodes tyrosine sulfotransferase (*AtTPST*), which plays a crucial role in the tyrosine sulfation of PSY and phytosulfokine (PSK) precursor polypeptides, both crucial for plant development. The *tpst-1* mutant plants displayed stunted roots, pale green leaves, and a dwarf phenotype due to defective endogenous PSY and PSK signaling. Exogenous application of sulfated peptides rescued the root defects in the *tpst-1* mutant, confirming the peptides’ biological activity.

**Figure 8:**
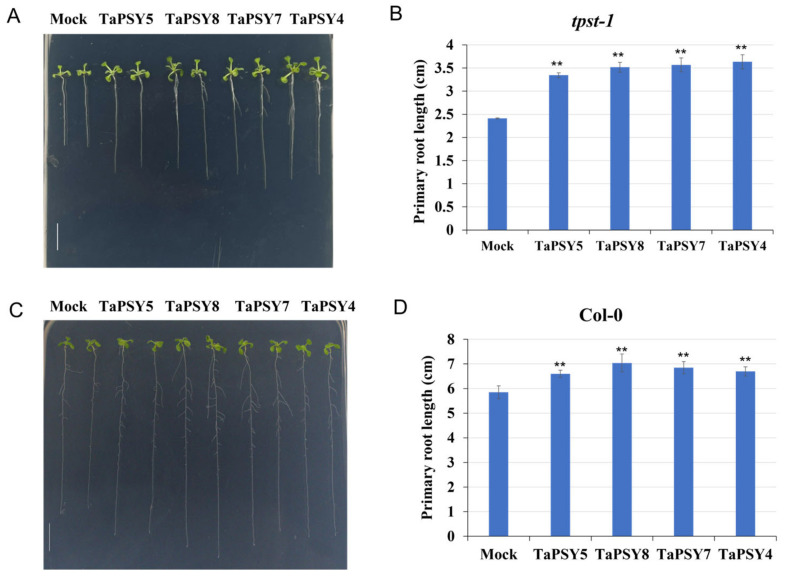
Sulfated TaPSY peptides promote root growth in *Arabidopsis*. (**A**,**C**) Representative images of 7-day-old Col-0 and *tpst-1* seedlings grown vertically on 1/2 MS plates, supplemented with or without 500 nM of the indicated peptides. (**B**,**D**) Root lengths of wild-type *Arabidopsis* and the *tpst-1* mutant as shown in (**A**,**C**), respectively. Error bars represent ± SE. Scare bar = 10 mm. Statistically significant differences were determined by one-way ANOVA. ** *p* < 0.01.

The *tpst-1* seedlings were grown on MS plates for 3 d, after which seedlings with similar root lengths were transferred to plates supplemented with 500 nM of TaPSY4, TaPSY5, TaPSY7, or TaPSY8 peptides, respectively. After 7 d, the length of the primary roots was measured. Treatment with 500 nM of these peptides resulted in a 37% increase in root elongation in *tpst-1* plants compared to mock treatment, highlighting their role in promoting root growth (Figure 8A,B). Additionally, treatment of wild-type plants with TaPSY peptides enhanced primary root growth and increased lateral root formation compared to untreated seedlings (Figure 8C,D).

**Figure 9:**
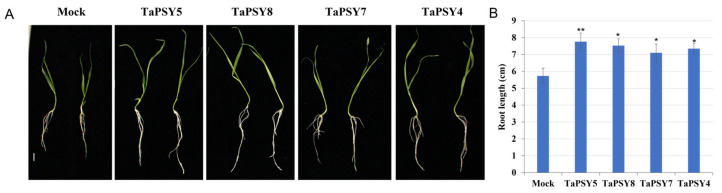
Sulfated TaPSY peptides promote root growth in wheat variety Fielder. (**A**) Representative images showing root lengths of 7-day-old seedlings of Fielder grown on 0.5×MS plates, 0.3% sucrose, with or without 1 μM of the indicated peptides (*n* ≥ 20). (**B**) The average seedling root length treated with TaPSY peptides for 7 d. Error bars indicate mean ± SE. Scare bar = 10 mm. Statistically significant differences were determined by one-way ANOVA. ** *p* < 0.01 and * *p* < 0.05.

We also investigated the physiological effect of TaPSY peptides on wheat root growth. Our findings showed that the exogenous application of 1 μM sulfated TaPSY peptides significantly increased root length in the wheat variety Fielder. Notably, the synthetic TaPSY5 peptide significantly enhanced root growth compared to control seedlings. The average root length of untreated seedlings was 57 mm, while seedlings treated with the TaPSY5 peptide showed an average root length of 78 mm, demonstrating TaPSY5’s pronounced effect on promoting primary root growth in wheat (Figure 9A,B).

### 2.8. TaPSY5-Meditated Root Growth in Wheat Is Associated with Hydrogen Peroxide Accumulation

**Figure 10:**
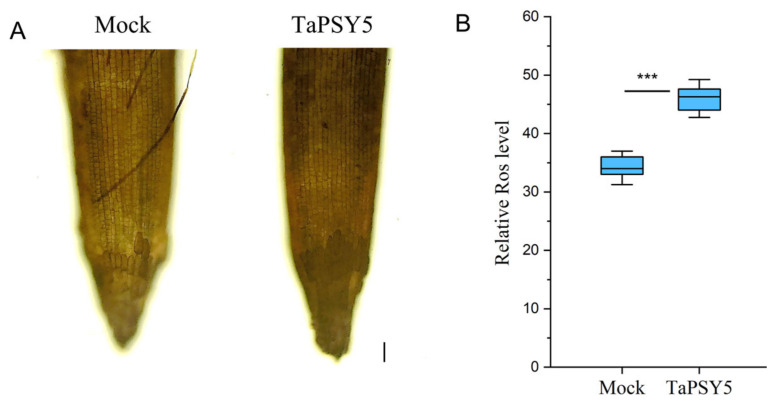
TaPSY5 peptide promotes H_2_O_2_ accumulation in wheat taproots. (**A**) Representative images of ROS levels in wheat roots treated with 1 μM of TaPSY5 peptide or mock for four days. (**B**) Quantification of ROS levels. *N* = 15. Significant difference was obtained using a one-way ANOVA test. *** *p* < 0.001. Error bars represent mean ± SD. Scale bar = 50 μm.

To explore whether reactive oxygen species (ROS) play a role in TaPSY5-mediated root growth in wheat, we assessed ROS levels in wheat roots using 3,3’-diaminobenzidine (DAB) staining. Wheat roots treated with the TaPSY5 peptide exhibited higher levels of ROS accumulation compared to untreated plants (Figure 10A,B). This suggests that the TaPSY5 peptide influences ROS levels in wheat taproots.

### 2.9. Overexpression of TaPSY10 Promotes Root Growth in Arabidopsis Thaliana

**Figure 11:**
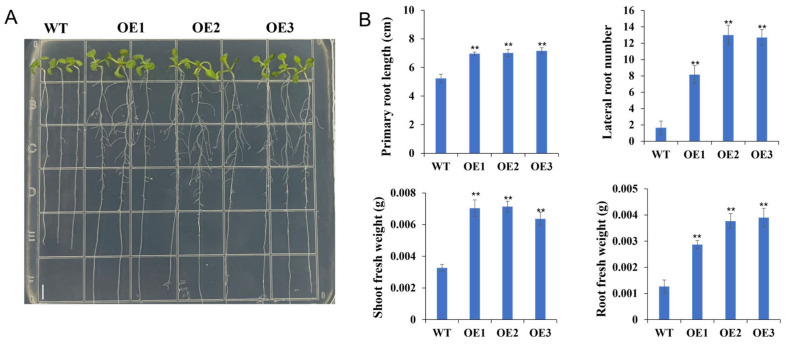
Overexpression of *TaPSY10* promotes *Arabidopsis* root growth. (**A**) Overexpression of *TaPSY10* increases primary root length. Growth of *Arabidopsis* wild-type (WT) and *TaPSY10-OE* seedlings were grown on 1/2 MS agar plates with 1% sucrose for 7 d. (**B**) Measurement of root length in the WT, and three independent *TaPSY10-OE* transgenic lines (TaPSY10#OE1, TaPSY10#OE2, and TaPSY10#OE3). Error bars indicate standard error. *N* = 20–25 seedlings. Statistical significance compared to the WT was determined using an ANOVA test. ** *p* < 0.01. Scale bar = 5 mm.

Since *TaPSY10* was highly expressed in root tissue compared to other *TaPSY* family members (Figure S5), we selected it for further study. To investigate its role in root development, we generated *Arabidopsis* transgenic lines overexpressing *TaPSY10* under the control of the constitutive CaMV 35S promoter. Phenotypic analysis of three homozygous overexpression lines revealed that transgenic lines exhibited significantly accelerated root growth compared to the wild-type plants. Primary root lengths in *TaPSY10* overexpression lines TaPSY10-OE1, TaPSY10-OE2, and TaPSY10-OE3 increased by 32%, 33%, and 36%, respectively, relative to the wild-type (Figure 11A). Additionally, transgenic lines exhibited a higher total number of lateral roots and larger cotyledons compared to the wild-type seedlings. Furthermore, transgenic plants showed significantly increased shoot fresh weight by 115%, 118%, and 95%, respectively, and root fresh weight by 126%, 197%, and 207%, respectively, compared to the control plants (Figure 11B). These findings suggest that the *TaPSY10* gene plays a crucial role in regulating primary and lateral root development.

### 2.10. Overexpression of TaPSY10 Positively Regulates Genes Related to Root Morphogenesis

**Figure 12:**
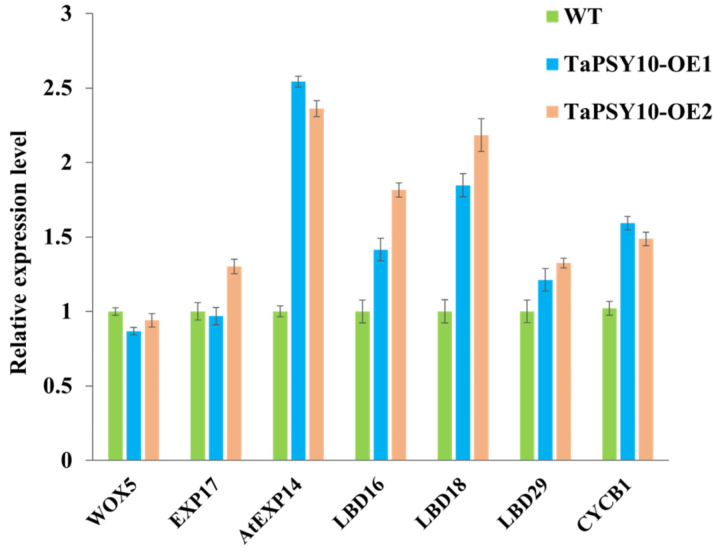
Relative expression levels of root morphogenesis-related genes in the WT and *TaPSY10*-overexpression (OE) lines by qRT-PCR. *Arabidopsis Atactin2* was used as an internal reference. Values are presented as mean ± SD.

Several genes involved in lateral root formation, such as *LATERAL ORGAN BOUNDARIES DOMAIN* (*LBDs*), *EXPANSIN* (*EXP*), and cell cycle genes (*CYCB1*), are known to regulate root development in *Arabidopsis*. To explore the potential role of *TaPSY10* in root morphogenesis, we conducted a qRT-PCR analysis to examine gene expression changes in roots of the WT and *TaPSY10* overexpression lines. The results showed that the expression levels of *LBD16*, *LBD18*, and *EXP14* were significantly upregulated in the transgenic lines compared to the WT, while *LBD29* expression showed a slight increase. These findings suggest that *TaPSY10* overexpression may influence lateral root development by modulating the expression of genes related to root morphogenesis (Figure 12).

## 3. Discussion

In plants, numerous small peptides, each consisting of fewer than 100 amino acids, play diverse roles in plant growth, development, stress responses, and defense responses [8]. Most of these peptides are derived from non-functional precursor proteins containing an N-terminal signal peptide. These precursors undergo post-translational modifications and enzymatic processing to produce biologically active peptides [22]. Among these, AtPSY1, identified in *Arabidopsis* cell culture media, is a disulfated peptide that promotes cell expansion and division [13,23]. The PSY family peptides are critical regulators of plant growth and development [24]. Researchers have identified PSY precursor genes in a few species, including nine in *Arabidopsis* and seven in rice [15]. In this study, we identified 29 genes encoding TaPSY family protein precursors at the whole-genome level in wheat, surpassing the number found in other species [15,20]. Wheat is composed of three subgenomes (A, B, and D), derived from two hybridization and polyploidization events, accompanied by tandem duplication, segmental duplication, and transposition events [25,26]. This expansion in gene copy number is likely due to genome duplication during wheat polyploidy, resulting in the duplication of some homologous genes. We observed that *TaPSY1* is exclusive to the B and D subgenomes, with its homolog in the A genome absent, possibly due to evolutionary loss or genome assembly quality. Evolutionary analysis revealed that wheat and rice PSY precursors are distributed across different subgroups, whereas *Arabidopsis* PSY members cluster mainly in subgroups V and VI, suggesting that wheat PSY genes are more closely related to those of rice.

Multiple sequence alignment of the full-length TaPSY precursors revealed that the C-terminal regions of wheat PSY homologs are conserved among different species, resembling the AtPSY1 domain and the RaxX sequence [13,17]. The conserved five amino acid residues (DY, N, H, and P) in the C-terminal region, represented as DYXXXX[AP]NXXHXP, are consistent with those found in PSY precursors of *Arabidopsis*, rice, and maize [15], indicating functional conservation among these homologs. Furthermore, our results revealed that a conserved Asp-Tyr (DY) site exists within the wheat PSY domain. Similar findings have been reported in *Arabidopsis* [20], identifying the mature peptides AtPSY2, AtPSY3, AtPSY5, AtPSY6, and AtPSY8 in *Arabidopsis*, where peptides range in length from 14 to 21 amino acids and begin with the conserved Asp-Tyr, aligning with our results. The conserved Asp-Tyr residues are essential for PSY peptide function, as the tyrosine sulfation (DY) site is crucial for biological activity, catalyzed by TPST (AtTPST) in *Arabidopsis* [27,28]. TPST is also responsible for sulfonylating PSK and RGF precursors, producing mature tyrosine-sulfated peptides [13,29]. We speculate that the potential Asp-Tyr sites in TaPSY peptides undergo post-translational modification via TPST. The absence of sulfation might reduce biological activity, lowering the affinity of the unsulfated peptide for the PSYR1 receptor [30].

The PSY family not only regulates root development but also plays key roles in abiotic stress responses [20]. In this study, RNA-seq and qRT-PCR analyses revealed distinct expression patterns among *TaPSY* genes. *TaPSY9-5A/5B/5D* and *TaPSY6-3A/3B/3D* were predominantly expressed in stems, while *TaPSY3*, *TaPSY4*, *TaPSY5*, *TaPSY8*, and *TaPSY10* exhibited high expression levels in roots. This suggests that *TaPSY* genes serve specialized roles in root and stem development. Additionally, *TaPSY8* and *TaPSY10* were downregulated in response to drought stress and ABA treatment, indicating their potential involvement in negatively regulating drought tolerance.

There is evidence supporting the role of PSY peptides in root growth [8,31]. The exogenous application of mature AtPSY5 peptide in *Arabidopsis* has been found to promote plant growth and development [20]. Previous studies have demonstrated that synthetic RaxX peptide (HVGGGDY(SO_3_H)PPPGANPKHDPPPR), derived from *Xoo* and AtPSY1(DY(SO_3_H)GDPSANPKHDPGVPPS), activates primary root growth in *Arabidopsis* [13,17]. The *tpst-1* mutant of *Arabidopsis*, which lacks functional *PSY* family members, exhibits a pleiotropic phenotype with shorter roots, pale-green leaves, dwarfism, and fewer reproductive organs [32,33]. This mutant serves as a model to demonstrate the effects of the exogenous application of sulfated peptides. Recent studies have identified PSY-like peptides from root-knot nematode (MigPSY1, MigPSY2, and MigPSY3), significantly increasing root length in *tpst-1*, and confirming their PSY peptide-like activity [32]. Our study builds on this by examining wheat TaPSY peptides. We synthesized TaPSY4, TaPSY5, TaPSY7, and TaPSY8 peptides based on alignment with AtPSY orthologs and RaxX21-sY from the *Xanthomonas* strain [17,18]. These peptides lacked C-terminal hydroxy- and L-Ara_3_-modifications. Our results showed that 500 nM concentrations of these peptides induced primary root growth in both wild-type and *tpst-1* mutants, aligning with previous findings on AtPSY1 and RaxX peptides. The TaPSY peptides likely require tyrosine sulfation for full activity, mimicking AtPSY1 in function. These peptides also promote root growth in wheat, with the TaPSY5 peptide showing the most significant effect. Recent research has demonstrated that AtPSY peptides act as ligands for AtPSYR receptors (AtPSYR1, AtPSYR2, and AtPSYR3) [20,31]. We hypothesize that wheat PSY peptides bind to similar receptors to modulate root development, although further study is required to identify them.

Root development is tightly linked to ROS and nitric oxide, which regulate root elongation, lateral root formation, and root hair development [34,35]. Previous studies have shown a relationship between root growth and ROS levels [36]. ROS are predominantly distributed in the elongation zone and are involved in maintaining root growth [37]. In *Brassica rapa* L., certain CEP peptides have been found to mediate root growth through a ROS-dependent manner [38]. Our DAB staining results revealed that TaPSY5 peptide treatment increased H_2_O_2_ levels in wheat roots, suggesting that TaPSY5 influences root development through ROS production.

To further explore the role of *TaPSY* precursor peptide genes in root development, we focused on *TaPSY10* due to its notably high expression levels in roots. Overexpression of *TaPSY10* in *Arabidopsis* led to an increase in both lateral root numbers and primary root length, indicating its positive regulatory role. Previous studies have shown that transcription factors LBD16, LBD18, and LBD29 are critical for lateral root initiation [39]. *LBD18*, for example, regulates genes involved in cell wall loosening, such as *EXP14*, by binding directly to its promoter, thereby promoting lateral root development. Additionally, cell cycle-related genes like *CYCB1* are involved in establishing a lateral root-inducible system [40]. Our findings revealed that overexpressing *TaPSY10* upregulated the expression of *CYCB1*, *LBD16, LBD18*, and *EXP14*, suggesting that *TaPSY10* modulates these genes to regulate root development. Overall, our study highlights the crucial roles of both synthetic PSY peptides and *TaPSY10* overexpression in root development. Further studies involving wheat transgenesis and phenotypic analyses will be necessary to fully elucidate the function of *TaPSY* genes.

## 4. Materials and Methods

### 4.1. Identification of the TaPSY Gene Family

To identify the potential PSY peptide homologs, the full-length PSY precursor sequences from *Arabidopsis* and rice were downloaded from the *Arabidopsis* Information Resource (TAIR) (https://www.arabidopsis.org/, accessed on 2 December 2022) and the UniProtKB (http://www.uniprot.org, accessed on 26 December 2022) database. These sequences were used as queries in a BLASTP search against the wheat genome database (http://202.194.139.32/, accessed on 8 October 2022). To be classified as a member of the wheat PSY family, candidates needed to meet the following criteria: an expect value (E-value) of ≤ 20, sequence lengths between 60 and 200 amino acids, and the presence of the DYXXXX[AP]NXXHXP motif at the C-terminal region. Sequences lacking the Asp-Tyr start in the PSY-like motif were excluded. All identified TaPSY homologs were aligned with PSY proteins from *Arabidopsis* and rice, and the proteins exhibiting a similar PSY-like motif were confirmed as TaPSY proteins. The molecular weight (kDa) and isoelectric point (pI) of TaPSY proteins were calculated using the ExPASy tool (https://web.expasy.org/compute_pi/, accessed on 1 August 2022). Signal peptides were predicted using the TargetP 2.0 server (http://www.cbs.dtu.dk/services/SignalP/, accessed on 4 February 2023). Domain features of TaPSY proteins were visualized using WebLogo (http://weblogo.berkeley.edu/logo.cgi, accessed on 25 January 2023).

### 4.2. Chromosome Location, Sequence Alignment, and Phylogenetic Analysis

The chromosomal locations of the *TaPSY* genes were extracted from the Ensembl Plants database (https://plants.ensembl.org/Triticum_aestivum/Info/Index, accessed on 13 March 2023) and mapped with TBtools software (https://github.com/CJ-Chen/TBtools, accessed on 5 August 2022) [41]. The PSY precursor sequences from wheat, rice, and *Arabidopsis* were aligned using the ClustalX 2.01 program with default parameters (http://www.clustal.org/clustal2/, accessed on 19 May 2023), and a phylogenetic tree was constructed using MEGA 7.0 software (http://megasoftware.net/, accessed on 8 July 2023) with the maximum likelihood (ML) method under the LG model, with 1,000 bootstrap replicates. Visualizations were created using the EvolView online tool (https://evolgenius.info//evolview-v2/#login, accessed 6 January 2023).

### 4.3. Gene Structure and Cis-Element Analysis

The cDNA and genomic sequences of the *TaPSY* genes were obtained from the Ensembl Plants database (https://plants.ensembl.org/Triticum_aestivum/Info/Index, accessed on 22 February 2022). The distribution of exons and introns was visualized using the Gene Structure Display Server (GSDS2.0, http://gsds.cbi.pku.edu.cn/, accessed on 7 April 2023). The upstream sequences (2000 bp) of the transcriptional start site of the *TaPSY* genes were retrieved from WheatOmics 1.0 (http://202.194.139.32/, accessed on 12 September 2023) and then submitted to the Plant CARE database (http://bioinformatics.psb.ugent.be/webtools/plantcare/html/, accessed 9 December 2023) for *cis*-acting element prediction.

### 4.4. Analysis of Wheat PSY Gene Replication

MCScanX (https://github.com/wyp1125/MCScanx, accessed on 12 July 2023) was used to determine the number of duplication events according to a previous study [42]. The duplicated *TaPSY* gene pairs were visualized using the Advance Circos package in TBtools version 2.119 [43]. KaKs Calculator 2.0 was used to calculate the non-synonymous substitution rate (Ka) and the synonymous substitution rate (Ks) for each gene pair [44].

### 4.5. Expression Analysis of TaPSY Genes Using RNA-Seq Datasets

Expression profiles of *TaPSY* genes were examined using RNA-seq data from the Wheat Expression Browser (http://202.194.139.32/expression/wheat.html, accessed 5 May 2024) across various tissues and under abiotic stress. Tissue-specific expression levels in roots, stems, leaves, spikes, and seeds at three different developmental stages were analyzed using RNA-seq data (accession number: ERP004714). The expression patterns of *TaPSY* genes in leaves under drought, heat, and their combination, and salt treatments, were analyzed using RNA-seq data (accession number: SRP045409) as reported in a previous study [45]. The expression data were normalized and converted to log_2_ TPM (transcripts per million mapped reads) values. Heatmaps were generated using TBtools (version 2.119) [43].

### 4.6. Quantitative Real-Time PCR (qRT-PCR) Analysis of TaPSY Genes

The wheat cultivar Chinese Spring was planted in a controlled greenhouse for qRT-PCR analysis. Tissue samples were collected from roots, leaves, and stems at the seedling stage, spikes at flowering, and grains at 5 and 15 days after anthesis (DAA). For stress treatments, seeds were sterilized with 10% sodium hypochlorite (NaClO) for 10 min, washed at least five times, and germinated in Petri dishes at 22 °C in the dark. Seedlings were cultured with Hoagland nutrient solution for two weeks in a growth chamber with a 16/8 h light/dark cycle, 25/20 °C day/night temperature, and 60% humidity. They were treated with half-strength Hoagland nutrient solution containing 100 mM IAA, 100 μM abscisic acid (ABA), and 20% polyethylene glycol (PEG6000) for 0, 1, 3, 6, 12, and 24 h. Root samples were flash-frozen in liquid nitrogen and stored at −80 °C for RNA extraction. Total RNA was extracted using a Plant RNA Kit (Omega Bio-Tek, Guangzhou, China) and reverse transcribed with the ReverTra Ace^®^ qPCR RT Master Mix (Toyobo, Osaka, Japan) according to the manufacturer’s instructions. qRT-PCR was performed on a QuanStudio™ 5 (Applied Biosystems, Foster City, CA, USA) using KOD SYBR qPCR Mix (TOYOBO, Osaka, Japan) in a 20 µL reaction volume containing 10 µL of 2× KOD SYBR qPCR Mix, 0.4 μL of each primer (10 μM), 1 μL of cDNA template, and 8.2 μL of ddH_2_O. All primers used for the qRT-PCR are listed in Table S1. The *TaGAPDH* gene was used as a reference for tissue analysis and *TaActin1* for stress treatment. Relative expression levels of selected *TaPSY* genes were calculated using the 2^-∆∆Ct^ method [46], with three biological replicates for each experiment.

### 4.7. Synthesis of the TaPSY Peptides

The C-terminal PSY-like sequences were identified based on sequence alignment with AtPSY proteins [20]. The natural processing and modifications of these peptides were unknown. Chemically synthesized tyrosine-sulfated peptides, including TaPSY4 (DY(SO_3_H)PGSGPNDRHTPKAPGT), TaPSY5 (DY(SO_3_H)PRYGANGRHNPEGPHP), TaPSY7 (DY(SO_3_H)GGANSRHDPRRRPGRNG), and TaPSY8 (DY(SO_3_H)PGSSANGRHEPPRSPGRG), were cobtained from DGpeptide Company (http://www.dgpeptides.com/) with a purity higher than 90%. The synthetic peptides lacked hydroxy- and L-Ara_3_-modifications. All peptides were dissolved in distilled water to a 1 mM concentration and stored at −20 °C to avoid freeze–thaw cycles.

### 4.8. Plant Material, Growth Conditions, and Root Length Quantification

The *Arabidopsis* plants used in this experiment were derived from the Col-0 ecotype background. The T-DNA insertion line *tpst-1* (SALK_009847) was previously described [47]. Homozygous mutants were isolated from the progeny and identified through PCR amplification using gene- and T-DNA-specific primers as outlined previously [26]. For root growth assays, peptides or water (mock) were added to a final concentration of 500 nM before pouring into the plates. Seeds of the wild-type and *tpst-1* mutants were surface-sterilized with sodium hypochlorite for 10 min, rinsed five times, and sown on 90 mm square plates (8 seeds per plate) containing half-strength Murashige and Skoog (MS) medium with 1% sucrose (pH 5.7, 0.8% agar). Plates were incubated vertically at 23 °C for 3–4 d. Seedlings with similar root lengths were then transferred to Petri dishes supplemented with 500 nM peptides (three plates per treatment) and grown vertically in a growth chamber under a 16 h light/8 h dark cycle at 22 ± 1 °C. Root lengths were measured after 7 days.

For the exogenous application of wheat TaPSY peptides, seeds of the variety ‘Fielder’ were sterilized with 70% ethanol for 1 min, followed by 0.1% HgCl_2_ for 10 min. The seeds were sown on 0.8% agar plates containing half-strength MS medium and grown for three days. Once roots reached 1 cm in length, the endosperm was removed to prevent nutrient interference. Subsequently, seedlings with uniform root lengths were transferred to plates containing half-strength MS medium, 0.5% agar, 0.5% sucrose, and 1 μM of each of the indicated peptides. Root lengths were measured in at least 20 plants after seven days of incubation at 25 °C under a 16 h light/8 h dark cycle, with humidity maintained at 65–70%. Results were consistent across three independent experiments.

### 4.9. H_2_O_2_ Detection

To quantify H_2_O_2_ levels in wheat roots, a DAB staining kit (3,3′-diaminobenzidine, Bioworld Technology, Inc., DB5038, Louis Park, MN, USA) was used according to the manufacturer’s instructions. Briefly, wheat roots treated with 1 μM TaPSY5 peptide or mock (ddH_2_O) for 4 d were stained with 1× DAB buffer in the dark for 3 min, followed by three 3 min rinses with 1× PBS buffer. Root tips were placed on slides and observed under an Echo Revolve inverted fluorescent microscope (RVL-100-G, ECHO, San Diego, CA, USA). Three independent biological replicates were performed with 15 seedlings per treatment. The level of H_2_O_2_ was quantified using ImageJ software (http://imagej.nih.gov/ij/, accessed on 5 July 2023).

### 4.10. Phonotypic Analysis of TaPSY10 Overexpression Lines in Arabidopsis

The full-length cDNA sequence of the *TaPSY10* gene was amplified from Fielder root samples and cloned into the pCAMBIA1300 vector using *Knp*I and *Pst*I (New England Biolabs, NEB, Ipswich, MA, USA) restriction enzymes to generate the overexpression vector *35S::TaPSY10*. The resulting plasmid was introduced into *Agrobacterium tumefaciens* strain GV3101 and used to transform *Arabidopsis* via the floral dip method. Positive T_3_ transgenic lines were selected for analysis.

Seeds from both transgenic *Arabidopsis* and wild-type plants were surface-sterilized with 2% bleach for 10 min, washed five times with sterile water, and sown on MS medium. After three days of growth, seedlings with similar root lengths were transferred to 0.5× MS medium supplemented with vitamins (MSP09; Caisson Labs, Smithfield, VA, USA), 1% sucrose (pH 5.7), and 0.5% Phytagel (P8169; Sigma, St Louis, MO, USA) for 6 d. Primary root lengths were measured and analyzed using SPSS 19.0 software (SPSS Corp, Chicago, IL, USA).

## 5. Conclusions

We identified 29 *TaPSY* genes in wheat, categorizing them into six subgroups. The PSY peptides share a highly conserved motif at the C-terminus across multiple plant species. The qRT-PCR analysis revealed that most *TaPSY* genes exhibit higher expression levels in roots and stems, along with diverse expression patterns in response to ABA, IAA, drought stress, and salt stress. Synthetic sulfated TaPSY peptides were shown to stimulate root growth in both wild-type *Arabidopsis* and *tpst-1* mutant plants, indicating their bioactive role as small peptides. Furthermore, the synthetic sulfated TaPSY5 peptide significantly enhanced primary root growth in wheat by influencing the ROS levels. Overexpression of the *TaPSY10* gene resulted in increased primary root length and a greater number of lateral roots, highlighting the role of these peptides in promoting wheat development. In summary, our findings provide valuable insights into the role of these active small peptides in promoting wheat root development.

## Data Availability

All data, tables, and figures are included in this manuscript or Supplementary Materials.

## Supplementary Materials

The following supporting information can be downloaded at: https://www.mdpi.com/article/10.3390/ijms252312663/s1.

## Abbreviations

PSY	Plant peptide containing sulfated tyrosine
PSY1R	PSY RECEPTOR1
PSK	Phytosulfokine
TPST	Tyrosine protein sulfotransferase
RGF1	Root growth meristem factors
PTMs	Post-translational modifications
MS	Murashige and Skoog
ABA	Abscisic acid
EXP	EXPANSIN
CYCB1	Cell cycle
LBD	Lateral organ boundaries domain
DAB	3,3′-diaminobenzidine
ROS	Reactive oxygen species
* Xoo *	* Xanthomonas oryzae * pv. *oryzae*
H_2_O_2_	Hydrogen peroxide
