# Supplementary material for: Functional Characterization of Plant Peptide-Containing Sulfated Tyrosine (PSY) Family in Wheat (Triticum aestivum L.)
Source: Int J Mol Sci. 2024 Nov 25;25(23):12663. doi: 10.3390/ijms252312663 (PMC11641228; doi:10.3390/ijms252312663)
Supplement: Supplementary file 1 [file ijms-25-12663-s001.zip › Supplementary Figures.pdf]

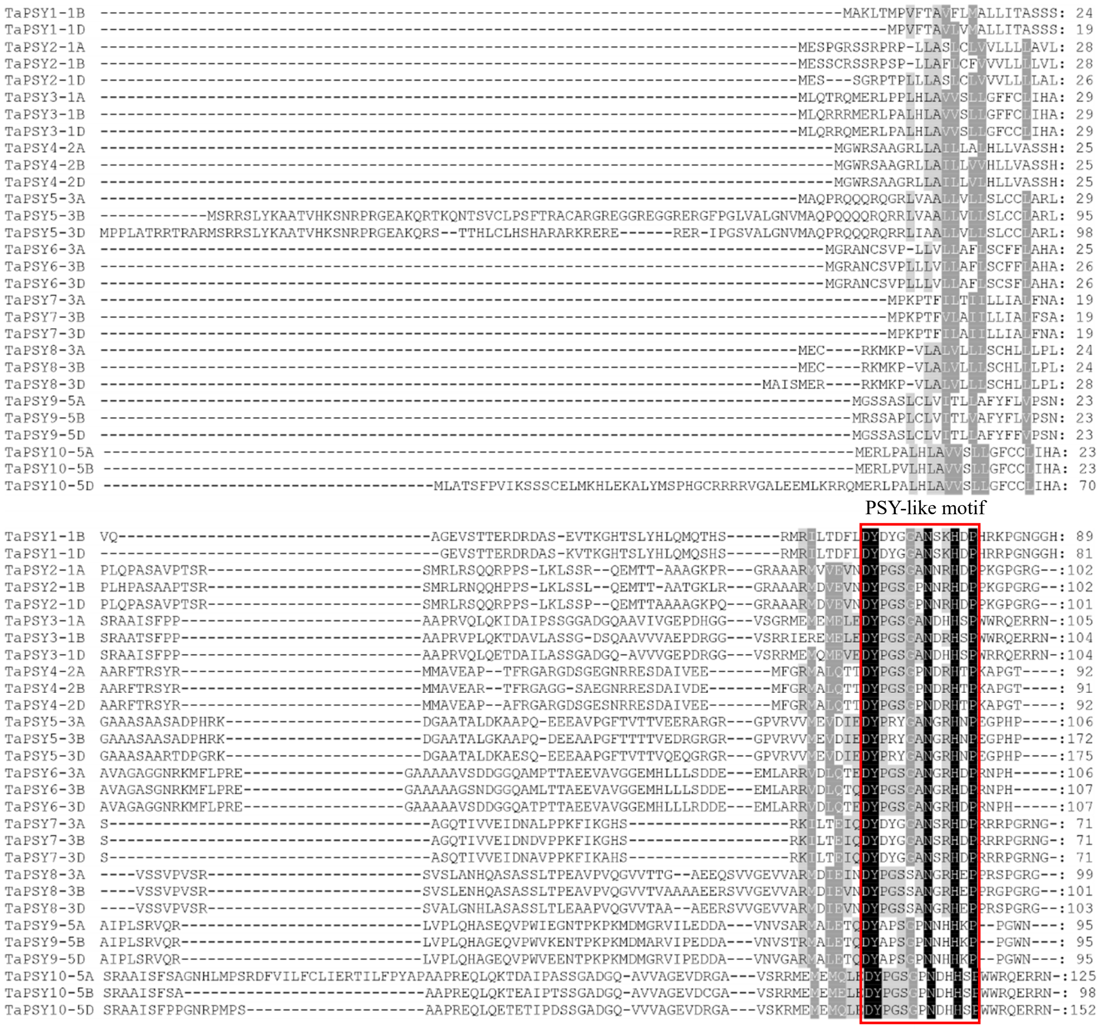

Figure S1. Alignment of predicted TaPSYs amino acid sequences with a signature PSY-like motif in the C-terminal region.

	Hormone													Abiotic/biotic stress															Development						
	P-box	ABRE	ABRE3a	ABRE4	TGA-element	CGTCA-motif	TGACG-motif	GARE-motif	TCA-element	AAGAA-motif	CCAAT-box	AuxRR-core	ERE	MYB-like sequence	MYB	Myb-binding site	MYB recognition site	STRE	MYC	MBS	LTR	DRE1	DRE core	TC-rich repeats	WUN-motif	W box	ARE	WRE3	O2-site	GCN4_motif	CAT-box	RY-element	circadian	CCGTCC motif	HD-Zip 1
TaPSY1-1B		1			1					1					5	3		3	1				4		1	1	3	1	1		1	1		1	
TaPSY1-1D		4	1	1		3	3							1	4		1	5	3	1			1		2	1		1			2	2			
TaPSY2-1A		1			1	1	1				1				5	3	1	1	3	1	1		1			1	3		1					1	1
TaPSY2-1B	1	4				2	2		1		3				3		3	4	3	2		1				2	2					1		3	
TaPSY2-1D	1	3	1	2	1	3	3			1	1			1	5	3	1	3	3	1	2					2			1		1			2	
TaPSY3-1A		2			1	4	4			1					3	1		4	1	1	1		2				1	2	1	1	1			2	
TaPSY3-1B		6	2	3	1	2	2				1		1	2	7		1	2	2	2				1		1	2		2	3	1				
TaPSY3-1D		3	1	1		3	3							1	4			4	1	2	3		1			1	1		1	2	3		1	1	
TaPSY4-2A		5	1	1		2	2							2	5				3				1		2	1	1	1	1						
TaPSY4-2B		3			1	4	4				1	1	3	1	4	1	1	1	4				1	1		1	1	1							
TaPSY4-2D		4	1	1	1	5	5		1		1			1	4		1		2		1			1		2	2	1							
TaPSY5-3A		3	2	2											1			5	8				1			1	1	1	2		1			1	
TaPSY5-3B		4			2	6	6						2	1	3			6	4		3		1			1	1	1	1		1				
TaPSY5-3D		2			1	1	1	1			1			1	3	1	1	2	8		1		2			1	1	3						1	
TaPSY6-3A		7	1	1		3	3	1	1	1		1			4	3		6	2			2	1			2		2			2			1	
TaPSY6-3B		1	3	3		1	1					2	2	1	3	1		3	3		1	1				1					1	1			
TaPSY6-3D		3	1	1		2	2		1						2	2			4			1	2			1		3			1			1	
TaPSY7-3A	2	4	1	1					1			1	2	1	11	5		1	6	2	1					4	3		1		1				
TaPSY7-3B	1	3	1	1				1	1			1	1	1	15	6		1	6	3	1					5	3		1		1				
TaPSY7-3D	2	3							1			1	2	1	1	4		1	6	2	1					4	3		1		1				
TaPSY8-3A	1				1				1	3		1			1			2	6				1			1	1	1						4	
TaPSY8-3B		4			1	1	1	1	1	1	2	1			4	2	2	3	5	2			2		1	3		1						3	
TaPSY8-3D		2			1	2	2		1					1	4			6	1	1			1			1	1	1			1			3	
TaPSY9-5A		3							1			1			5	2		4	5	1	1		3	1			1	1	1					1	
TaPSY9-5B		3	1	1		1	1		1	1				1	9	2		4	7	2	1			1			1	2	1						
TaPSY9-5D		3							1						5	2		2	5	1	1			2			1	2	1					1	
TaPSY10-5A							2								8	2		3	1	2								2	2	1	1	2			
TaPSY10-5B							2			1				1	5	1		4	4	1	1			1			2	2	3		1				
TaPSY10-5D	1						3			1	1				7	1	1	6	5	2							1	1	1		1				

Figure S2. Prediction of cis-acting regulatory elements in the *TaPSY* gene promoters.

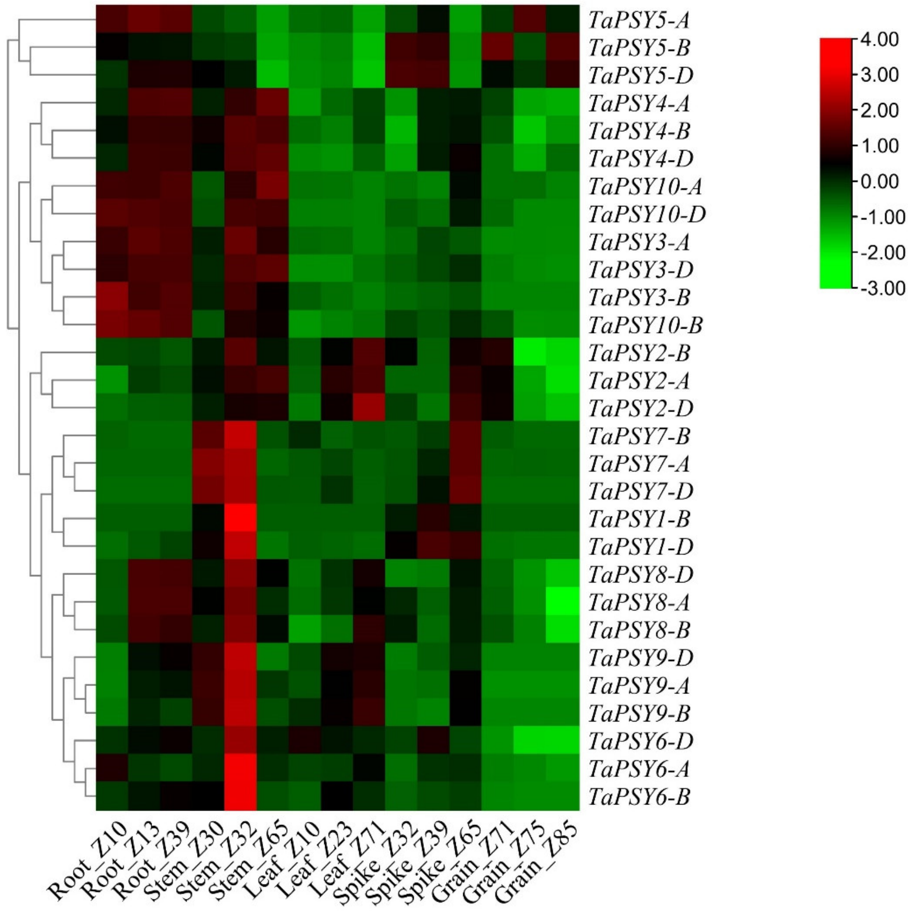

Figure S3. Heatmap of *TaPSY* gene expression patterns in five different tissues at three different developmental stages. The RNA-sequence data was obtained from leaves, stems, roots, spikes, and grains at developmental stages. For each tissue, three biological replicates were used. The color gradient (red/black /green) indicates the gene expression level (from high to low).

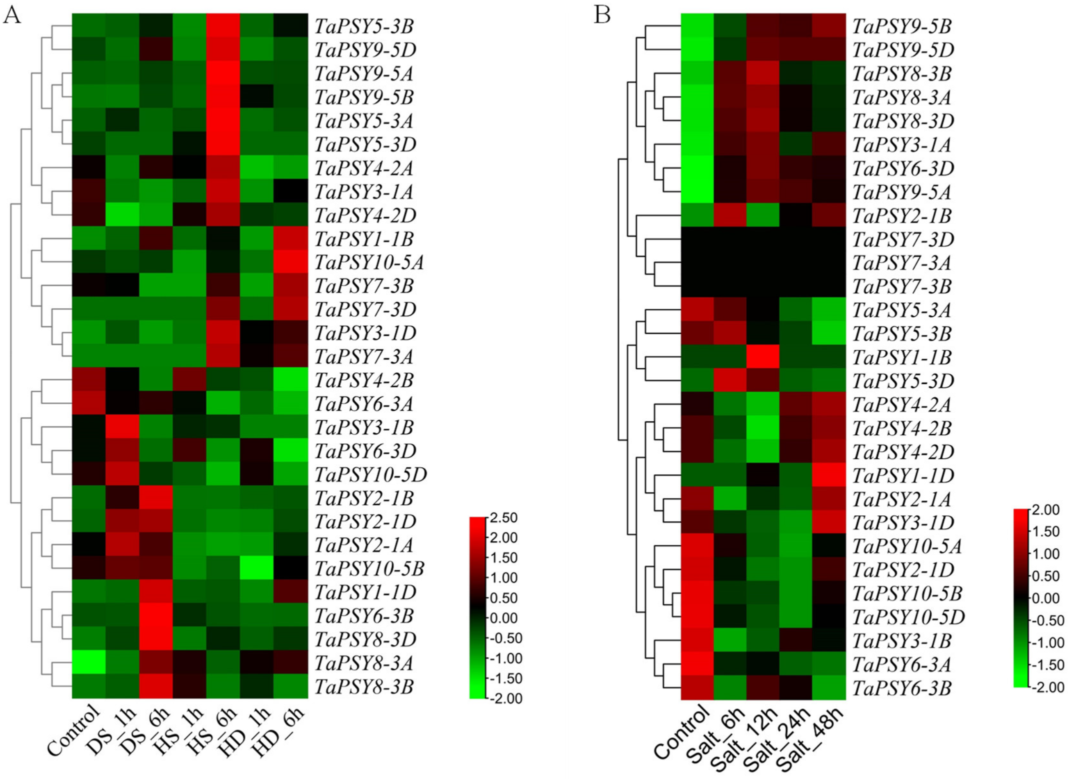

Figure S4. Expression patterns of *TaPSY* genes under drought and salt treatments. The heat maps generated by TBtools show the cluster map of *TaPSY* genes under drought (**A**) and salt (**B**) treatments. Values are created using log2-transformed transcripts per million (TPM) expression values.

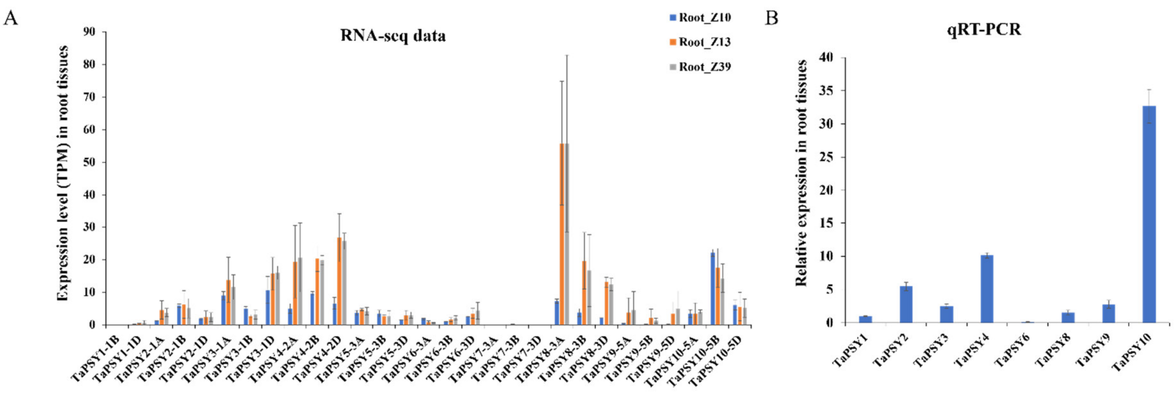

Figure S5. Gene expression of *TaPSYs* in root tissues. (**A**) The expression patterns of *TaPSY* genes in root tissue at different developmental stages using RNAseq data. (**B**) qRT-PCR analysis of selected *TaPSY* genes in root tissues at the seedling stage.
