# Supplementary material for: Functional Characterization of Plant Peptide-Containing Sulfated Tyrosine (PSY) Family in Wheat (Triticum aestivum L.)
Source: Int J Mol Sci. 2024 Nov 25;25(23):12663. doi: 10.3390/ijms252312663 (PMC11641228; doi:10.3390/ijms252312663)
Supplement: Supplementary file 1 [file ijms-25-12663-s001.zip › Supplementary Tables.pdf]

Table S1. Primer sequences used in this study

Gene name	Forward Primer	Reverse Primer
TaPSY1	GGATGCCTCCGAAGTGACAA	GGATCGTGTTTGGAGTTGGC
TaPSY2	CGCCGTCTCTGAAGCTTTC	GAGCTCGGTTCACCCTCTTC
TaPSY3	AATGGAGAGACTGCCTGCAC	GGGTAGTCCTCCACCTCCAT
TaPSY4	CTTCACGAGGAGCTACAGGA	ACTTTGACATGGCACGATC
TaPSY5	AGGACTATCCCCGGTATGG	GCGAACCAACCTAGCTGTAACT
TaPSY6	CTCTCCTGCTTCTTCCTCGC	TTCTGGCCAACATCTCCTCG
TaPSY7	AGCGGAGAACAACATGCCTA	TCTCTAGCCATTCCTCCCGG
TaPSY8	AGATGAAGCCCGTCCTTGC	ACTTCGCCGACGACGCTT
TaPSY9	GAACAACCACCACAAGCCAC	TGAGCCACGCCCAACTTTTA
TaActin1	AAATCTGGCATCACACTTTCTAC	GTCTCAAACATAATCTGGGTCATC
TaGAPDH	GACCCAGACAACTCGCAAC	GGAATCCATGACCACCTAC
LBD16	TACAACGGCGGGGACAGGT	AGCTGTGTCTTAGATCTCCG
LBD18	GTGCATAAAGTGTTCGGAGC	CTTCGTTGTTGCGTGGCCCA
LBD29-	GCTAGGCTTCAAGATCCCATC	TGTGCTGCTTGTTGCTTTAGA
EXP14	CAATACCGGAGAGTGGCTTGCC	TTGTTAGATATAACTGTACGGCC
EXP17	GACCTTTTATGGCGGAAGTGATGCC	TCTCGGTGGGTTGCACCAACCTCC
WOX5	AAGCTTGCGAAGAAGATTGTCAAGAGG	GATATCCGTGGTGGTCTCTCGAATATA
CYCB1	CTCAAAATCCCACGCTTCTTGTGG	CACGTCTACTACCTTTGGTTTCCC
AtACTIN2	GGCTCCTCTTAACCCAAAGGC	CACACCATCACCAGAATCCAG
35s::TaPSY10-5B	GGACGAGCTCGGTACC-ATGGAGAGGTTGCCTGTGCTG	GAACGAAAGCTCTGCAG-TCAATTCCTCCTCTCCTGCCG

Table S2. Basic information of the *TaPSY* genes identified in wheat

Gene name	Gene ID	Chromosome Location	Signal peptide (SP)	Protein length (aa)	Isoelectric point	Molecular mass (KDa)
TaPSY1-1B	TraesCS1B02G381100	613726905-613727702	27	89	7.02	9834.09
TaPSY1-1D	TraesCS1D02G369000	448238900-448239986	20	81	8.2	8956.04
TaPSY2-1A	TraesCS1A02G305300	497902190-497902938	35	102	11.8	10847.61
TaPSY2-1B	TraesCS1B02G316000	540365845-540366598	35	102	10.79	10970.69
TaPSY2-1D	TraesCS1D02G305000	402438050-402438819	33	101	10.81	10591.25
TaPSY3-1A	TraesCS1A02G394200	560314110-560314989	29	105	5.89	11497.07
TaPSY3-1B	TraesCS1B02G422500	645519077-645520000	29	104	9.97	11618.23
TaPSY3-1D	TraesCS1D02G402300	467625723-467626199	29	104	6.9	11489.07
TaPSY4-2A	TraesCS2A02G156700	103239429-103240678	26	92	10.19	9973.3
TaPSY4-2B	TraesCS2B02G182200	157265390-157266652	26	91	10.69	9832.17
TaPSY4-2D	TraesCS2D02G162500	106960751-106961927	26	92	10.19	10001.35
TaPSY5-3A	TraesCS3A02G180700	208454984-208456343	32	106	6.83	11307.78
TaPSY5-3B	TraesCS3B02G210400	246503573-246504813	–	172	10.43	18450.86
TaPSY5-3D	TraesCS3D02G185600	170930564-170931808	–	175	11.18	19355.04
TaPSY6-3A	TraesCS3A02G189500	233021585-233023278	25	106	4.98	11090.5
TaPSY6-3B	TraesCS3B02G218700	262078575-262079085	26	107	5.41	11205.68
TaPSY6-3D	TraesCS3D02G193000	183135201-183135877	26	107	5.19	11182.59
TaPSY7-3A	TraesCS3A02G253100	474435139-474436170	21	71	9.98	7914.17
TaPSY7-3B	TraesCS3B02G285000	456553593-456554786	21	71	9.69	7873.08

TaPSY7-3D	TraesCS3D02G254000	355630346-355631141	21	71	9.98	7914.17
TaPSY8-3A	TraesCS3A02G338900	585566784-585568154	27	99	6.26	10382.94
TaPSY8-3B	TraesCS3B02G370600	582751978-582753691	27	101	6.26	10579.19
TaPSY8-3D	TraesCS3D02G332500	444712770-444714612	31	103	8	10779.52
TaPSY9-5A	TraesCS5A02G228600	444595369-444596616	24	95	6.24	10436
TaPSY9-5B	TraesCS5B02G227400	403855427-403856622	24	95	7.95	10586.23
TaPSY9-5D	TraesCS5D02G239000	347276295-347277514	24	95	5.81	10451.96
TaPSY10-5A	TraesCS5A02G298600	505624255-505624748	26	125	6.18	13856.86
TaPSY10-5B	TraesCS5B02G297900	480298820-480299482	26	98	5.46	10731.13
TaPSY10-5D	TraesCS5D02G305400	400293478-400294173	–	152	8.44	17034.6

Table S3. Ka/Ks ratios of segmentally duplicated *TaPSY* genes

Gene name	Gene name	Ka	Ks	Ka/Ks	Duplicate event
TaPSY1-1B	*TaPSY1-1D*	0.027551	0.110971	0.248274	Segmental repeat
TaPSY2-1A	*TaPSY2-1B*	0.080678	0.062212	1.296832	Segmental repeat
TaPSY2-1A	*TaPSY2-1D*	0.048016	0.037508	1.28017	Segmental repeat
TaPSY2-1B	*TaPSY2-1D*	0.065049	0.031784	2.046562	Segmental repeat
TaPSY3-1A	*TaPSY3-1B*	0.083169	0.104335	0.797137	Segmental repeat
TaPSY3-1A	*TaPSY3-1D*	0.061709	0.178173	0.346342	Segmental repeat
TaPSY3-1B	*TaPSY3-1D*	0.100377	0.205659	0.488078	Segmental repeat
TaPSY4-2A	*TaPSY4-2B*	0.03018	0.158063	0.190937	Segmental repeat
TaPSY4-2A	*TaPSY4-2D*	0.01473	0.212215	0.069413	Segmental repeat
TaPSY4-2B	*TaPSY4-2D*	0.03533	0.140671	0.251156	Segmental repeat
TaPSY7-3A	*TaPSY7-3B*	0.031517	0.127969	0.246288	Segmental repeat
TaPSY7-3A	*TaPSY7-3D*	0.025081	0.040414	0.620597	Segmental repeat
TaPSY7-3B	*TaPSY7-3D*	0.031451	0.128888	0.24402	Segmental repeat
TaPSY8-3A	*TaPSY8-3B*	0.02803	0.094401	0.296923	Segmental repeat
TaPSY8-3A	*TaPSY8-3D*	0.047597	0.121614	0.391379	Segmental repeat
TaPSY8-3B	*TaPSY8-3D*	0.033115	0.051138	0.647568	Segmental repeat
TaPSY9-5A	*TaPSY9-5B*	0.047334	0.046168	1.025253	Segmental repeat
TaPSY9-5A	*TaPSY9-5D*	0.028106	0.061731	0.4553	Segmental repeat
TaPSY9-5B	*TaPSY9-5D*	0.037714	0.061731	0.610939	Segmental repeat
